# Statin-Induced Increase in HDL-C and Renal Function in Coronary Heart Disease Patients^§^

**DOI:** 10.2174/1874192400701010008

**Published:** 2007-07-28

**Authors:** Vasilios G Athyros, Anna I Kakafika, Athanasios A Papageorgiou, Efstathios D Pagourelias, Savvas D Savvatianos, Moses Elisaf, Asterios Karagiannis, Konstantinos Tziomalos, Dimitri P Mikhailidis

**Affiliations:** 1 Atherosclerosis and Metabolic Syndrome Units, 2nd Propedeutic Department of Internal Medicine, Aristotelian University, Hippocration Hospital, 49 Konstantinoupoleos St, Thessaloniki 546 42, Greece; 2 Department of Internal Medicine, Medical School, University of Ioannina, Greece; 3 Department of Clinical Biochemistry, (Vascular Prevention Clinic) Royal Free Hospital, Royal Free and University College Medical School, Pond Street, London NW3 2QG, UK

**Keywords:** Renal function, chronic kidney disease, dyslipidaemia, statins, high density lipoprotein, and coronary heart disease

## Abstract

**Background:**

Little is known about the potential of statin-induced high-density lipoprotein cholesterol (HDL-C) increase to improve renal function in coronary heart disease (CHD) patients.

**Methods and Results:**

In this*post hoc*analysis of the GREek Atorvastatin and Coronary heart disease Evaluation (GREACE) Study we investigated the effect of HDL-C increase after statin treatment on renal function. From a total of 1,600 patients, 880 were on various statins (mainly atorvastatin) and 720 were not. Other secondary prevention therapies were similar in the 2 groups. After a 3 year follow up, the lipid profile was unchanged in the statin untreated group and estimated glomerular filtration rate (eGFR) was reduced by 5.1% compared with baseline (P<0.0001). In contrast, in the statin treated group non-HDL-C was reduced by 43%, HDL-C was increased by 7% and there was a significant increase in eGFR compared with baseline by 9.8% (P<0.0001). In multiple regression analysis, the mean 7% increase in HDL-C in the treated arm during the entire study was associated with a 5.6% increase in eGFR recorded after the 6^th^ week of treatment. The odds ratio of eGFR increase with every 5% statin-induced rise in HDL-C was 1.78 (95% confidence interval 1.19-3.34; P=0.001).

**Conclusions:**

Statin treatment significantly improved renal function. Statin-induced HDL-C increase significantly and independently contributed to this improvement. This finding supports the concept that improving lipid variables other than low density lipoprotein cholesterol is also beneficial to preserving renal function.

## INTRODUCTION

Chronic kidney disease (CKD), including mild renal impairment, is recognized as an independent predictor of cardiovascular disease (CVD) in high-risk patients [1–3]. Similarly, in the general population there seems to be a graded association between a reduced glomerular filtration rate (GFR) and the risk of death, CVD events and hospitalization [4].

Dyslipidaemia is an independent risk factor for both CVD and CKD. The Physicians’ Health Study reported that from 4,483 participating healthy men with a normal baseline renal function, those with low high-density lipoprotein cholesterol (HDL-C) and high non-HDL-C [low-density lipopro-tein cholesterol (LDL-C) + very LDL-C (VLDL-C) + intermediate density lipoprotein cholesterol (IDL-C)] levels had double the risk for CKD after adjusting for other risk factors [5]. A recent study also showed that high apolipoprotein B and non-HDL-C levels were associated with an increased risk for deterioration of renal function in patients with CKD [6]. The issue is whether or not hypolipidaemic drug treatment is able to reverse this process

The results of the Helsinki Heart Study [7] and the Veterans Affairs High-Density Lipoprotein Intervention Trial (VA-HIT) [8] showed that fibrates might not exert a clinically relevant effect on rates of kidney function loss in individuals with low HDL-C and elevated non-HDL-C. The Heart Protection Study (HPS) showed that allocation to sim-vastatin significantly attenuated the fall in estimated-GFR (eGFR) compared with placebo [9]. A pooled analysis of 3 pravastatin survival trials also showed a decreased deterioration of renal function with pravastatin compared with placebo [10]. In the GREek Atorvastatin and Coronary heart disease Evaluation (GREACE) Study, statin treatment significantly increased eGFR by 12% and reduced serum uric acid levels, whereas renal function deteriorated in usual care patients [11–13]. Of note, the GREACE trial [11–13] showed that statin-induced improvement of eGFR was more evident in patients with mild renal impairment and contributed to the reduction in CVD events (multivariate analysis). A *post hoc* analysis of the Treating to New Targets (TNT) trial showed that over the 5 year study period there was a significant by 5.6% and 8.4% increase in eGFR with both doses of atorvas-tatin (10 and 80 mg/day, respectively) instead of the expected decline of about 5 mL/min/1.73 m^2^ [14]

However, it is not yet clear whether the relatively small increases in HDL-C observed during statin therapy contribute to the improvement in renal function, beyond the established beneficial effect of LDL-C reduction and non-HDL-C. In the present *post hoc* analysis of GREACE we address this issue.

## STUDY POPULATION - METHODS

**Study design, patients and methods.** The design of the GREACE study and its main findings have been previously described [11,12,15,16]. Briefly, GREACE included men (78%) and women (22%) with established coronary heart disease (CHD), aged <75 years (mean 58.3 years). Their serum LDL-C concentration was >100 mg/dL (2.6 mmol/L) and serum triglyceride (TG) levels <400 mg/dL (4.5 mmol/L). All patients attended the Atherosclerosis or the Metabolic Syndrome Units of the Hippocration University Hospital, Thessaloniki, and if eligible were randomized either into the structured care group, followed up by the University Clinic, or into the usual care group followed up by heart specialists or general practitioners of the patient’s choice outside the hospital. In the structured care group, the starting dose of atorvastatin was 10 mg/day. With evaluations every 6 weeks the dose of atorvastatin was titrated up to 80 mg/day for patients not reaching the National Cholesterol Education Program Adult Treatment Panel III (NCEP ATP III) LDL-C goal <100 mg/dL (2.6 mmol/L) with lower dosages. Patients in the usual care group were treated according to their physician’s standard of care. Usual care consisted of lifestyle changes plus necessary drug treatment, including lipid-lowering agents. Atorvastatin was not excluded from the usual care group. After dose titration patients were followed for a mean 3 year period with visits every 6 months. Serum creatinine (SCr) was measured using the Jaffé method (Olympus Diagnostica GmbH, Clare, Ire-land); normal range 0.6-1.3 mg/dL (55-115 μmol/L). eGFR was calculated using the Modification of Diet in Renal Disease (MDRD) formula [17]

The study subjects were randomized either to structured care (n=800) with dose titration of atorvastatin to achieve the LDL-C target of <100 mg/dl (2.6 mmol/L) or to usual care (n=800) that was followed by physicians of their choice. In this later group only 12% of subjects were on statin treatment and only 3% were at LDL-C target. In the present *post hoc* analysis we divided participants into those on statin and those without statin treatment, irrespective to their initial allocation to structured or usual care groups. Patients with SCr levels above the reference range [>1.3 mg/dL (115 μmol/L)] were excluded from the study.

**Classification of CKD.** In the Kidney Disease Outcomes and Quality Initiative (K/DOQI) guidelines [3], CKD is defined according to the presence or absence of kidney damage and level of kidney function, irrespective of diagnosis. In this classification scheme including 5 stages, stage 1 is associated with a normal eGFR (≥90 ml/min/1.73 m^2^), stage 2 with kidney damage and mildly decreased eGFR (60-89 ml/min/1.73 m^2^), stage 3 with moderately decreased eGFR (30-59 ml/min/1.73 m^2^), stage 4 with severely decreased eGFR (15-29 ml/min/1.73 m^2^) and stage 5 with an eGFR < 15 ml/min/1.73 m^2^.

**Statistical Analyses**. Treatment-based analyses of all patients according to statin or no statin treatment were performed. On study eGFR values were compared with those at baseline using ANOVA to assess differences over time within and between treatment groups. A univariate analysis was initially performed, including 25 predictors of renal function deterioration. Then, after removal of 6 predictors with a P > 0.10, 19 predictors were included in a multivariate Cox Predictive Model, involving backward stepwise logistic regression. All predictors were recorded as categorical factors (0-1). All univariate or multivariate analyses were performed with an HDL-C by 4 mg/dl (0.1 mmol/L) stepwise increase or reduction from baseline. Data were expressed as mean values. Standard deviations are presented in both Tables 1 and 2. The SPSS 11.01 software package (SPSS, Inc., Chicago, IL) was used for all statistical analyses. A two-tailed P < 0.05 was considered significant.

## RESULTS

Baseline characteristics of patients are shown in Table 1.

### 1 Renal Function at Baseline

According to K/DOQI, 642 patients had a stage 1, 864 a stage 2, and 94 a stage 3 renal function status. Patients from each renal function status were similarly distributed in the 2 analysed groups (Table 1). At baseline, eGFR in both analysed groups [with (n=880) or without (n=720) statin treatment] was 72 mL/min/1.73 m^2^ and 73 mL/min/1.73 m^2^, respectively (P = not significant)

### 2 Effect of Dyslipidaemia and Statin Treatment on eGFR

**Fig. (1):**
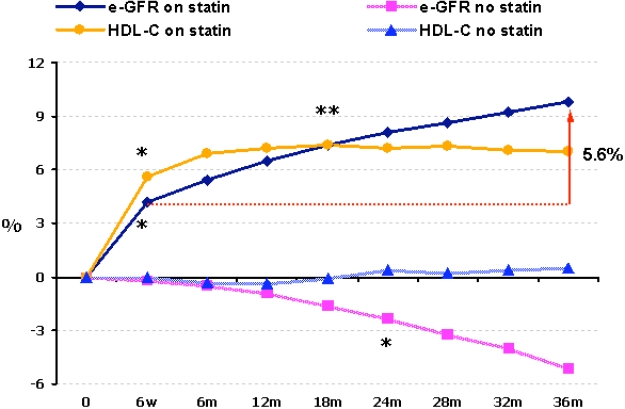
Time-course of changes of HDL-C and eGFR in statin treated and in statin untreated patients ^*^ P<0.0001 *vs* baseline ^ **^P<0.0001 *vs* 6th treatment week

**A. No statin treatment** (n=720). The 88% of the usual care group and those on atorvastatin that could not tolerate the drug formed this control group. There was a significant by 5.1% reduction in eGFR (P<0.0001 *vs* baseline) at the end of the 3 year period (Fig. 1).

**B. Statin treatment** (n=880). From patients allocated to a statin, 808 were on atorvastatin (mean dose 23 mg/day), 41 were on simvastatin (mean dose 20 mg/day), 23 on pravas-tatin (mean dose 24 mg/day) and 8 on fluvastatin (mean dose 40 mg/day). These patients presented an increase in eGFR levels by 4.2% at the 6^th^ week of treatment (P<0.001 *vs* baseline) and by 9.8% at the end of the study (P<0.0001 *vs* both baseline and 6^th^ week of treatment) (Fig. 1). The net increase in eGFR between 6^th^ week of treatment and the 3^rd^ year of treatment was 5.6% (Fig. 1). This increase was dependent on baseline eGFR levels. Patients with a eGFR < 77 mL/min/1.73 m^2^ (median value of all patients) had a mean increase in eGFR of 12.9% (P<0.0001), while patients with an eGFR > 77 mL/min/1.73 m^2^ had a mean increase in eGFR of 4.5% (P=0.002). Thus, the greatest benefit from statin treatment was observed in those with early renal dysfunction.

### 3 Lipid Profile at Baseline

Patients from both treatment groups had high total cholesterol, LDL-C, non-HDL-C and TG levels (Table 2). HDL-C was 39-40 mg/dl (1.0 mmol/l). Thus, the dyslipidaemia of the GREACE study could be characterized mostly as combined hyperlipidaemia.

### 4 Effect of Statin Treatment on the Lipid Profile

The lipid profile in the untreated group remained essentially unchanged throughout the study (Table 2). In the statin treated group, 97% of patients (n=854) had mean LDL-C levels < 100 mg/dL (2.6 mmol/L) after the titration period (mean change -46%) and 98% of patients (n=898) had mean non-HDL-C levels < 130 mg/dL (3.4 mmol/L) after the titration period (mean change -43%). The mean increase in HDL-C was 7% (P<0.001 *vs* baseline and P=0.002 *vs* on-study values in the usual care group). The LDL-C/HDL-C ratio was reduced by 49% (P<0.0001 *vs* both baseline and usual care), while the mean reduction in TGs was 30% (P<0.0001 *vs* baseline).

### 5 Effect of HDL-C Increase on eGFR

Multiple regression analysis showed that the renal function improvement (estimated by eGFR increase) in patients receiving statins was associated with a reduction in athero-genic lipoprotein levels as well as with the statin-induced increases in HDL-C (7% from baseline), over and above secondary CVD prevention therapies. This effect of HDL-C was seen in patients who achieved LDL-C levels <100 mg/dl (2.6 mmol/L) and non-HDL-C levels <130 mg/dl (3.36 mmol/L).

### 6 Other Factors that may have Influenced the Improvement in Renal Function

There wre no significant differences between the statin treated and untreated groups regarding demographic characteristics, CHD risk factors at baseline (Table 1) and concomitant drug treatment (especially in angiotensin converting enzyme inhibitors or calcium channel blockers that might influence eGFR) (Table 3). The levels of glycaemic control and both systolic and diastolic blood pressure during the study were similar between the 2 groups. Both at entry and during the study, smokers (4.4% and 3.7%, respectively) were similarly distributed in the 2 treatment groups. Similar numbers of patients lost >10% of their body weight in the 2 treatment groups (40 in the statin treated and 44 in the untreated group). Furthermore, the MDRD formula considers age, gender and race. Thus, it was highly unlikely that we had false low or false high eGFR values. Moreover, results were fully adjusted for 20 predictors of CHD related events (Tables 4 and 5). Thus, the beneficial effect on renal function should mainly be attributed to statin treatment. There were no extreme values of SCr at baseline [i.e. extremely high values, because patients were excluded from the study if they had a SCr value >1.3 mg/dl (115 μmol/l)]. The method used to assess SCr is reproducible and the use of mean values of a large number of patients together with the different effects on SCr in each group reduced to a minimum any regression to the mean effect. Moreover, there was no relation between HDL-C and serum uric acid levels if eGFR was taken into consideration. All patients in both treatment groups received advice on life-style changes and the body mass index (a rough index of compliance with life-style measures) was similar in both groups.

## DISCUSSION

We showd that renal function (represented by the eGFR) declines over 3 years in dyslipidaemic CHD patients with normal or mildly impaired renal function at baseline when they are not being treated with a statin. Statin treatment (mainly atorvastatin) not only inhibited this deterioration but significantly increased the eGFR in these patients. It has been proposed that CVD and renal disease progression are the consequences of the same underlying disorders [18]. Thus, CVD risk factor reduction strategies may also be beneficial in CKD [18,19]. Whether there are differences between statins in terms of renal benefit remains to be established [20].

In our tatin untreated patients, there was a gradual reduction in eGFR that became significant by the end of the 2^nd^ year of the study; renal function continued to deteriorate until the end of the study (a mean 3 year period). In statin treated patients, renal function improvement was manifest as early as the 6^th^ week of treatment; thereafter there was further improvement that continued until the end of the study. The early part of renal function improvement might be attributed to the pleiotropic effects of statins (e.g. anti-inflammatory effects and improved endothelial function) [21]. In support of these findings, when patients with CKD were given statins for CVD risk reduction, they showed evidence of improved renal function [22]. The improvement of renal function after the 6^th^ week of treatment might be related both to reduction of atherogenic lipoproteins and the increase of HDL-C.

High seum LDL-C has an adverse effect on glomerular mesangial cells and endothelial cells [23]. Moreover, in animal studies [24], elevated lipid content of VLDL and IDL played an important role in the pathogenesis of proteinuria and glomerulosclerosis. Clinical and experimental studies have demonstrated the role of lipoproteins in the decline of renal function with emphasis on glomerulosclerosis [25–28], neutrophil and macrophage infiltration [29] and upregulation of the cytokine interleukin (IL)-6 [30,31] or endothelial nitric oxide synthase [32]. Animal studies showed that the rate of decline of GFR is beneficially modified after improving the lipoprotein profile by dietary or pharmacological manipulation, including statins [28,33].

The preent study addressed whether or not the statin-induced HDL-C increase contributed to renal function improvement on top of LDL-C reduction and other secondary CVD prevention measures. HDL might improve renal func tion through several mechanisms [34]. Reverse cholesterol transport may contribute to the inhibition of intra-renal atherosclerosis and protect against direct toxic effects of lipids on renal cells [35]. Inhibiting the accumulation of lipopro-teins may also reduce LDL binding to receptors expressed by the mesangial cells and limit matrix production [36]. Another relevant mechanism might be the antioxidant effect of HDL [37]. However, can HDL particles protect from atherosclerosis or glomerulosclerosis in both low and high LDL-C states? Our data suggest that they can. It is also plausible that low LDL-C and anti-inflammatory effects of statins might improve HDL particle performance [39]. First, lower concentrations of LDL-C leave less cholesterol to be “scavenged” by HDL particles. Second, lowering oxidized LDL levels results in inhibition of growth factor receptor activation and of subsequent matrix metalloproteinase upregulation [37,38]. Another mechanism of action of HDL results from the inhibition of the production, even at low LDL-C concentrations, of cellular reactive oxygen species (ROS) [37].

In the bsence of inflammation, HDL has a complement of antioxidant enzymes that work to maintain an anti-inflammatory state [39,40]. However, in the presence of systemic inflammation, these antioxidant enzymes can be inactivated and HDL can accumulate oxidized lipids and proteins that transform it to a proinflammatory particle [40]. This is probably the case for our statin untreated CVD patients.

Finally patients on statin treatment had fewer recurrent CVD events during the study, thus preserving cardiac performance and renal blood flow.

## STUDY LIMITATIONS

The stuy has all the restrictions of a *post hoc* analysis and there was no assessment of urine protein. In addition, we did not directly measure GFR; an effect of statins on creatin-ine secretion from renal tubular cells cannot be ruled out and might have affected eGFR without an actual effect on renal function.

## CONCLUSIONS

There ws a decline in renal function over a period of 3 years in CHD patients not treated with statins. In contrast, statin treatment significantly improved renal function. There was a contribution of statin-induced HDL-C increase (7%) to this improvement over and above low non-HDL-C levels and other potentially beneficial therapies. This evidence supports the concept that improving lipid variables other than LDL-C may be relevant for both CHD and CKD prevention.

## DECLARATION OF INTEREST

The present study was conducted independently; no Company or Institution supported it financially. Some of the authors have attended conferences and participated in advisory boards and other trials sponsored by various pharmaceutical companies

## Figures and Tables

**Table 1:** Baseline Characteristics of the Statin Treated and Untreated Groups

Parameter	Statin Treated (N=880)	Statin Untreated (N=7200)	P Value
Men/women (%)	79/21	78/22	NS
Age (years)	59±9	59±13	NS
CHD (%)	100	100	NS
Dyslipidaemia (%)	100	100	NS
Current smokers (%)	4.4	3.7	NS
History of diabetes mellitus (%)	20	19	NS
History of arterial hypertension (%)	43	42	NS
Congestive heart failure (%)	8	7	NS
Prior PCI/CABG (%)	35	36	NS
Recent hospitalization for CHD (%)	7	8	NS
Renal function K/DOQI			
Stage 1 (%)	41	40	NS
Stage 2 (%)	54	53	NS
Stage 3 (%)	5	7	NS

NS: not significant

CHD:coronary heart disease, PCI: percutaneous coronary intervention,CABG: coronary artery bypass graft, K/DOQI: Kidney Disease Outcomes and Quality Initiative.

**Table 2:** Lipid Parameters (Mean Value ± Standard Deviation) at Baseline and During the Study in the Statin Treated and Untreated Groups

Lipid Parameter	Statin Treated (n=880)		Statin Untreated (n=720)		Differences Between On-Study Values
	Baseline Value	Mean On-Study Value & % Change *vs* Baseline	Baseline Value	Mean On-Study Value & % Change *vs* Baseline	Statin *vs* No Statin P Value
Total cholesterol (mg/dl)	258±35	164±9 -36%†	256±39	244±40 -4%	<0.0001
LDL cholesterol (mg/dl)	181±23	99±3 -46%†	180±26	175±24 -3%	<0.0001
VLDL cholesterol (mg/dl)	37±11	24±5 -31%†	36±9	35±10 -2%	<0.0001
HDL cholesterol (mg/dl)	39±6	42±5 7% *	39±7	40±6 1%	0.002
Non-HDL cholesterol (mg/dl)	219±32	125±6 - 43%†	217±29	206±31 -5%	<0.0001
Triglycerides (mg/dl)	182±61	127±31 -30%†	179±59	173±64 -4%	<0.0001

^†^P< 0.0001 *vs* baseline.

^*^P< 0.01 *vs* baseline.

LDL: low density lipoprotein, VLDL: very low density lipoprotein, HDL: high density lipoprotein

To convert data from mg/dL to mmol/L divide total cholesterol, LDL cholesterol, VLDL cholesterol, HDL and non-HDL cholesterol values by 38.7; triglyceride values by 88.5.

**Table 3:** Medical Therapy During the Study

	Statin Treated N=880	Statin Untreated N=720	P Value
Aspirin or other antiplatelet agents (%)	87	86	NS
Beta-blockers (%)	85	84	NS
ACE inhibitors or ARBs (%)	54	53	NS
Nitrates (%)	14	15	NS
Calcium channel blockers (%)	26	27	NS
Diuretics (%)	11	12	NS
Statins (%)	100	0	< 0.0001

NS: not significant.

ACE: angiotensin converting enzyme, ARBs: Angiotensin II receptor blockers.

**Table 4:** Univariate Odds Ratios and 95% Confidence Intervals for eGFR Increase After the 6^th^Treatment Week

Variables	Univariate ORs and 95% CI for eGFR Increase	
	OR (95% CI)	P Value
Age (years)	0.47 (0.34-0.64)	<0.0001
Gender male	1.44 (1.07-1.93)	0.015
CVD event during the study	0.82 (0.65-1.17)	0.2
Use of beta-blockers during the study	0.67 (0.50-0.91)	0.02
Use of ACE inhibitors during the study	1.27 (0.95-1.02)	0.064
*On treatment non-HDL-C <130 mg/dL	5.60 (4.00-7.85)	<0.0001
HDL-C with every 5% increase	3.49 (2.53-4.82)	<0.0001

Fourteen univariate predictors of all CHD-related events all with p <0.10 (the above plus: current smoking, family history of premature CHD, history of hypertension, history of diabetes mellitus, prior revascularization, acute coronary syndrome, and prior myocardial infarction) were initially entered.

^*^On treatment values were considered as the mean values during the entire study.

To convert data from mg/dL to mmol/L divide non-HDL-C values by 38.7

OR: odds ratio, CI: confidence interval, CVD: cardiovascular disease, ACE: angiotensin converting enzyme, HDL-C: high density lipoprotein cholesterol.

**Table 5:** Multivariate Cox Predictive Model for eGFR Increase After the 6^th^ Week of Treatment (Backward Stepwise Logistic Re- gression)

Variables	Multivariate Cox Predictive Model for eGFR increase	
	OR (95% CI)	P value
Age (years)	0.79 (0.62-0.93)	0.02
Gender male	1.32 (1.14-1.65)	0.003
CVD event during the study	0.86 (0.64-1.37)	0.4
Use of beta-blockers during the study	0.82 (0.61-1.19)	0.2
Use of ACE inhibitors during the study	1.14 (0.84-1.04)	0.1
*On treatment non-HDL-C <130 mg/dL	3.12 (1.79-5.12)	<0.0001
HDL-C with every 5% increase	1.78 (1.19-3.34)	0.001

Fourteen univariate predictors of all CHD-related events all with p <0.10 (the above plus: current smoking, family history of premature CHD, history of hypertension, history of diabetes mellitus, prior revascularization, acute coronary syndrome, and prior myocardial infarction) were initially entered.

^*^On treatment values were considered as the mean values during the entire study.

To convert data from mg/dL to mmol/L divide non-HDL-C values by 38.7

OR: odds ratio, CI: confidence interval, CVD: cardiovascular disease, ACE: angiotensin converting enzyme, HDL-C: high density lipoprotein cholesterol.

## References

[1] Sarnak MJ, Levey AS, Schoolwerth AC (2003). Kidney disease as a risk factor for development of cardiovascular disease: a statement from the American Heart Association Councils on Kidney in Cardiovascular Disease, High Blood Pressure Research, Clinical Cardiology, and Epidemiology and Prevention. Circulation.

[2] Anavekar NS, McMurray JJ, Velazquez EJ (2004). Relation between renal dysfunction and cardiovascular outcomes after myocardial infarction. N Engl J Med.

[3] Kaplan RC, Heckbert SR, Furberg CD, Psaty BM (2002). Predictors of subsequent coronary events, stroke, and death among survivors of first hospitalized myocardial infarction. J Clin Epidemiol.

[4] Go AS, Chertow GM, Fan D, McCulloch CE, Hsu CY (2004). Chronic kidney disease and the risks of death, cardiovascular events, and hospitalization. N Engl J Med.

[5] Schaeffner ES, Kurth T, Curhan GC (2003). Cholesterol and the risk of renal dysfunction in apparently healthy men. J Am Soc Nephrol.

[6] Ozsoy RC, van der Steeg WA, Kastelein JJ, Arisz L, Koopman MG (2007). Dyslipidaemia as predictor of progressive renal failure and the impact of treatment with atorvastatin. Nephrol Dial Transplant.

[7] Mänttäri M, Tiula E, Alikoski T, Manninen V (1995). Effects of hypertension and dyslipidemia on the decline in renal function. Hypertension.

[8] Tonelli M, Collins D, Robins S, Bloomfield H, Curhan GC (2004). Effect of gemfibrozil on change in renal function in men with moderate chronic renal insufficiency and coronary disease. Am J Kidney Dis.

[9] Collins R, Armitage J, Parish S, Sleigh P, Peto R (2003). Heart Protection Study Collaborative Group: MRC/BHF Heart Protection Study of cholesterol-lowering with simvastatin in 5963 people with diabetes: A randomised placebo-controlled trial. Lancet.

[10] Tonelli M, Isles C, Craven T (2005). Effect of pravastatin on rate of kidney function loss in people with or at risk for coronary disease. Circulation.

[11] Athyros VG, Mikhailidis DP, Papageorgiou AA (2004). The effect of statins versus untreated dyslipidemia on renal function in patients with coronary heart disease: A subgroup analysis of the GREek Atorvastatin and Coronary heart disease Evaluation (GREACE) study. J Clin Pathol.

[12] Athyros VG, Elisaf M, Papageorgiou AA (2004). GREACE Study Collaborative Group: Effect of statins versus untreated dyslipidemia on serum uric acid levels in patients with coronary heart disease: a subgroup analysis of the GREek Atorvastatin and Coronary-heart-disease Evaluation (GREACE) study. Am J Kidney Dis.

[13] Athyros VG, Mikhailidis DP, Liberopoulos EN (2007). Effect of statin treatment on renal function and serum uric acid levels and their relation to vascular events in patients with coronary heart disease and metabolic syndrome: A subgroup analysis of the GREek Atorvastatin and Coronary heart disease Evaluation (GREACE) Study. Nephrol Dial Transplant.

[14] Shepherd J, Wenger N (2006). Intensive lipid lowering with atorvastatin is associated with a significant improvement in renal function: The Treating to New Targets (TNT) Study. American College of Cardiology. Scientific Sessions.

[15] Athyros VG, Papageorgiou AA, Mercouris BR (2002). Treatment with atorvastatin to the National Cholesterol Educational Program goals versus usual care in secondary Coronary Heart Disease prevention. The GREek Atorvastatin and Coronary-heart-disease Evaluation (GREACE) Study. Curr Med Res Opin.

[16] Liberopoulos EN, Mikhailidis DP, Athyros VG, Elisaf MS (2006). The effect of cholesterol lowering treatment on renal function. Am J Kidney Dis.

[17] Levey AS, Bosch JP, Lewis JB, Greene T, Rogers N, Roth D (1999). A more accurate method to estimate glomerular filtration rate from serum creatinine: a new prediction equation. Ann Intern Med.

[18] Sarnak MJ, Levey AS (2000). Cardiovascular disease and chronic renal disease: A new paradigm. Am J Kidney Dis.

[19] Athyros VG, Karagiannis A, Liberopoulos EN, Elisaf M, Mikhailidis DP (2007). Statin treatment may be beneficial to both the kidneys and the heart. Perit Dial Int.

[20] Athyros VG, Karagiannis A, Kakafika A, Elisaf M, Mikhailidis DP (2007). Statins and renal function. Is the compound and dose making a difference?. Nephrol Dial Transplant.

[21] Tsiara S, Elisaf M, Mikhailidis DP (2003). Early vascular benefits of statin therapy. Curr Med Res Opin.

[22] Epstein M, Campese VM (2005). Pleiotropic effects of 3-hydroxy-3-methylglutaryl coenzyme a reductase inhibitors on renal function. Am J Kidney Dis.

[23] Gin H, Rigalleau V, Aparicio M (2000). Lipids, protein intake, and diabetic nephropathy. Diabetes Metab.

[24] Joles JA, van Goor H, van der Horst ML, van Tol A, Elema JD, Koomans HA (1995). High lipid levels in very low density lipoprotein and intermediate density lipoprotein may cause proteinuria and glome-rulosclerosis in aging female analbuminemic rats. Lab Invest.

[25] Guijarro C, Kasiske BL, Kim Y, O’Donnell MP, Lee HS, Keane WF (1995). Early glomerular changes in rats with dietary-induced hyper-cholesterolemia. Am J Kidney Dis.

[26] Keane WF, Kasiske BL, O’Donnell MP (1988). Hyperlipidemia and the progression of renal disease. Am J Clin Nutr.

[27] Diamond JR, Karnovsky MJ (1988). Focal and segmental glomerulosclerosis: analogies to atherosclerosis. Kidney Int.

[28] Kasiske BL, O’Donnell MP, Cleary MP, Keane WF (1988). Treatment of hyperlipidemia reduces glomerular injury in obese Zucker rats. Kidney Int.

[29] O’Donnell MP, Kasiske BL, Kim Y, Schmitz PG, Keane WF (1993). Lovastatin retards the progression of established glomerular disease in obese Zucker rats. Am J Kidney Dis.

[30] Yokota N, O’Donnell M, Daniels F (2003). Protective effect of HMG-CoA reductase inhibitor on experimental renal ischemia-reperfusion injury. Am J Nephrol.

[31] Bolton CH, Downs LG, Victory JG (2001). Endothelial dysfunction in chronic renal failure: roles of lipoprotein oxidation and pro-inflammatory cytokines. Nephrol Dial Transplant.

[32] Joyce M, Kelly C, Winter D, Chen G, Leahy A, Bouchier-Hayes D (2001). Pravastatin, a 3-hydroxy-3-methylglutaryl coenzyme A reductase inhibitor, attenuates renal injury in an experimental model of ischemia-reperfusion. J Surg Res.

[33] Keane WF, Kasiske BL, O’Donnell MP (1987). The role of lipids in progressive glomerular disease. Adv Exp Med Biol.

[34] Fielding CJ, Fielding PE (1995). Molecular physiology of reverse cholesterol transport. J Lipid Res.

[35] Moorhead JF, Chan MK, El-Nahas M, Varghese Z (1982). Lipid nephro-toxicity in chronic progressive glomerular and tubulointerstitial disease. Lancet.

[36] Abrass CK (2004). Cellular lipid metabolism and the role of lipids in progressive renal disease. Am J Nephrol.

[37] Robbesyn F, Auge N, Vindis C (2005). High-density lipoproteins prevent the oxidized low-density lipoprotein-induced epidermal [corrected] growth factor receptor activation and subsequent matrix metalloproteinase-2 upregulation. Arterioscler Thromb Vasc Biol.

[38] Auge N, Garcia V, Maupas-Schwalm F, Levade T, Salvayre R, Negre-Salvayre A (2002). Oxidized LDL-induced smooth muscle cell proliferation involves the EGF receptor/PI-3 kinase/Akt and the sphin-golipid signalling pathways. Arterioscler Thromb Vasc Biol.

[39] Navab M, Berliner JA, Subbanagounder G (2001). HDL and the inflammatory response induced by LDL-derived oxidized phos-pholipids. Arterioscler Thromb Vasc Biol.

[40] Navab M, Anantharamaiah GM, Reddy ST, Van Lenten BJ, Ansell BJ, Fogelman AM (2006). Mechanisms of disease: proatherogenic HDL-an evolving field. Nat Clin Pract Endocrinol Metab.

